# Global Psychological Distress and Risk of Atrial Fibrillation Among Women: The Women's Health Study

**DOI:** 10.1161/JAHA.112.001107

**Published:** 2012-06-22

**Authors:** William Whang, Karina W. Davidson, David Conen, Usha B. Tedrow, Brendan M. Everett, Christine M. Albert

**Affiliations:** Department of Medicine, Columbia University Medical Center, New York, NY (W.W., K.W.D.); University Hospital, Basel, Switzerland (D.C.); Division of Preventive Medicine, Brigham and Women's Hospital, Boston, MA (B.M.E, C.M.A.); Center for Arrhythmia Prevention, Division of Preventive Medicine and Cardiovascular Division Brigham and Women's Hospital, Boston, MA (U.B.T, C.M.A.)

**Keywords:** epidemiology, arrhythmia, women, atrial fibrillation

## Abstract

**Background:**

Symptoms of psychological distress and depression have been associated with risk of ventricular arrhythmias and sudden cardiac death. Their relationship with atrial arrhythmias, however, is less well studied.

**Methods and Results:**

We sought to assess the long-term relations between psychological distress and risk of atrial fibrillation (AF) in the Women's Health Study of female health professionals. We measured psychological symptoms with the Mental Health Inventory-5. Incident AF was assessed annually and verified through medical records. Among 30 746 women without history of cardiovascular disease or AF, 771 cases of AF occurred during a median follow-up of 125 months (interquartile range, 117–130 months). Global psychological distress was not associated with AF risk in age-stratified (*P*=0.61 for linear trend) or multivariable proportional-hazards models that included antidepressant use (*P*=0.34). A proxy measure for depression, consisting of Mental Health Inventory-5 score <53, antidepressant use, or both, was also not associated with AF risk in multivariable models (hazard ratio=0.99; 95% confidence interval, 0.78–1.25; *P*=0.93). In post hoc analyses of individual symptoms from the Mental Health Inventory-5, positive affect, “feeling happy some/a good bit of the time,” was associated with reduced risk of AF (hazard ratio=0.69; 95% confidence interval, 0.49–0.99; *P*=0.04); other depressive and anxious symptoms were not.

**Conclusions:**

In this prospective study of women without known cardiovascular disease, global psychological distress and specific depressive symptoms were unrelated to AF risk. **(*J Am Heart Assoc.* 2012;1:e001107 doi: 10.1161/JAHA.112.001107.)**

## Introduction

Several studies have suggested that depression and depressive symptoms are associated with ventricular arrhythmia and with sudden cardiac death.^1–5^ Atrial fibrillation (AF) is the most common sustained arrhythmia and confers increased risks of stroke and cardiac death.^6^ Although depressive symptoms have been associated with increased risk of death among patients with AF,^7^ data on the relationship with incident AF are relatively limited.^8,9^ The autonomic nervous system is known to influence the risk of AF, and abnormalities in markers of sympathetic and parasympathetic tone have been noted in affective disorders such as depression and panic disorder.^10,11^ Therefore, the autonomic nervous system may provide a mechanism by which AF is associated with psychological distress such as depressive symptoms and negative affect. In this study, we evaluated the relationship between a measure of global psychological distress, the Mental Health Inventory-5 (MHI-5), and long-term risk of incident AF among a large cohort of healthy female health professionals.

## Methods

All study subjects were participants in the Women's Health Study, a randomized trial of low-dose aspirin and vitamin E in the primary prevention of cardiovascular disease and cancer. Details of the study design have been published previously.^12^ Beginning in 1993, 39 876 female health professionals in the United States, who were ≥45 years of age and free of cardiovascular disease, cancer, or other major illnesses, were randomized to receive 100 mg aspirin every other day, 600 IU vitamin E every other day, both, or placebo. Randomized treatment ended on March 31, 2004, and women were invited to participate in continued observational follow-up, which was truncated for the present study at March 10, 2010. Of the original cohort, 4324 opted out of the observational follow-up study. These women were excluded from the present analysis because their AF could not be reliably confirmed.

On the 60-month questionnaire, global psychological distress was assessed with the MHI-5, a 5-item subscale of the Short-Form 36 health status survey.^13^ Each item asks respondents how much of the time over the past month (all, most, a good bit, some, little, or none) they felt nervous (anxiety), felt so down that nothing could cheer them up (depression), felt calm and peaceful (low affective activation), felt down and blue (depression), or felt happy (positive affect). The scale is scored from 0 to 100, with higher scores indicating greater psychological well-being. The MHI-5 instrument has been shown in samples based on the general population to have area under the receiver operating characteristic curve of 0.78 to 0.92 for mood disorders and 0.71 to 0.74 for anxiety disorders, compared with diagnoses based on psychiatric interviews.^14–19^ Antidepressant use was assessed on the 48-month questionnaire through an item that asked about any prescription medication use. Similar to prior studies that used this scale,^5,20^ we created 4 categories of MHI-5 score to indicate severity of global psychological distress: 86–100, 76–85, 53–75, and <53. We designated significant psychological distress according to MHI-5 score <53.^5,20^ Also, we created a proxy variable for clinical depression that consisted of MHI-5 score <53, reported antidepressant use, or both.^5^ In addition to our global assessment, we used individual items from the MHI-5 to assess for specific relationships between particular symptoms, particularly positive and negative affect, and risk of AF. Responses to the items were heavily skewed, and we grouped them into 3 categories for each symptom (all or most of the time, a good bit or some of the time, and little or none of the time).

Because not all data on cardiovascular risk factors were collected on the 60-month questionnaire, we obtained data on particular risk factors from the most recent questionnaire that inquired about them. Data on smoking and alcohol intake were collected from the 48-month questionnaire, and data on kilocalories from physical activity were collected from the 36-month questionnaire.

For the present analysis, we further excluded 2428 women who did not submit responses on the 60-month questionnaire. We also excluded 360 women with a history of cardiovascular disease, 854 women with a history of AF at the 60-month visit entry, and 1164 women with missing data for the MHI-5. The final study population consisted of 30 746 women.

Written informed consent was obtained from all participants. The study was approved by the institutional review boards of Brigham and Women's Hospital, Boston, MA, and Columbia University Medical Center, New York, NY.

Details about the confirmation of AF in the Women's Health Study have been reported previously.^21^ Women who enrolled in the continued observational follow-up study and who reported an incident AF event on at least 1 yearly questionnaire were sent an additional questionnaire to confirm the episode and to collect additional information. They also were asked for permission to review their medical records. For all deceased participants who reported AF during the trial and extended follow-up period, family members were contacted to obtain consent and additional relevant information. An end-point committee of physicians reviewed medical records for reported events according to predefined criteria. An incident AF event was confirmed if there was electrocardiographic evidence of AF or if a medical report clearly indicated a personal history of AF.

We computed proportions of participants with cardiovascular risk factors according to the baseline presence of affective symptoms, and comparisons were made with Mantel-Haenszel χ^2^ tests for trend for categorical measurements or with analysis of variance for continuous measurements. We used Cox proportional-hazards models to estimate age-stratified and multivariable-adjusted hazard ratios (HRs) for AF associated with MHI-5 category, using the date of return of the 60-month questionnaire as the starting point for follow-up. The proportional-hazards assumption was examined by including MHI-5 category by logarithm of time interaction terms in each model, and the assumption was found to be satisfied. We performed sensitivity analyses in which we reestimated all Cox models after censoring women at the date of their first cardiovascular event, defined by myocardial infarction, stroke, or coronary revascularization. We also performed analyses of individual symptoms on the MHI-5 and AF by estimating separate age-stratified Cox models, with frequency of the symptom as an ordinal variable (0 = reference, 1 = some or a good bit of the time, 2 = most or all of the time) and as a categorical variable.

## Results

Among 30 746 women without history of cardiovascular disease or AF, 2089 (6.8%) had significant global psychological distress, as represented by MHI-5 score <53 reported on the 60-month questionnaire (Table 1). Women with significant global distress were younger, had higher average body mass index, and had higher rates of diabetes and hypercholesterolemia. Current smoking was more frequent with worse symptoms of psychological distress, but alcohol intake was less frequent. Physical activity was lower among women with more severe symptoms.

**Table 1.** Coronary Heart Disease Risk Factors Among 30 746 Women Without Prior Cardiovascular Disease or AF According to MHI-5 Category

	MHI-5 Category			
	86–100 (Referent)	76–85	53–75	0–52
No. women (% of total)	11 834 (38.5)	10 696 (34.8)	6127 (19.9)	2089 (6.8)

Age,† mean±SD, y	60.4±7.2	58.6±6.6	57.7±6.2	56.9±6.0

Race, nonwhite, n (%)	506 (4.3)	462 (4.4)	297 (4.9)	83 (4.0)

Body mass index,† mean±SD, kg/m^2^	26.6±5.1	26.8±5.3	27.2±5.6	27.8±6.1

Diabetes,† n (%)	457 (3.9)	415 (3.9)	280 (4.6)	132 (6.3)

High cholesterol,† n (%)	5212 (44.0)	4741 (44.3)	2875 (46.9)	1022 (48.9)

Hypertension,† n (%)	4784 (40.4)	4327 (40.5)	2611 (42.6)	996 (47.7)

Current smoker,† n (%)	951 (8.1)	1025 (9.7)	713 (11.8)	334 (16.3)

Antidepressant medication, n (%)	439 (3.7)	685 (6.4)	643 (10.5)	430 (20.6)

Alcohol intake, ≥1 drink per day,† n (%)	1390 (11.9)	1194 (11.3)	617 (10.2)	208 (10.2)

Kilocalories of exercise, >1000 kcal/wk,† n (%)	4334 (36.6)	3491 (32.6)	1791 (29.2)	604 (28.9)

Because of missing data, the total number of observations was lower for race (n=30 435), body mass index (n=30 634), alcohol intake (n=30 406), smoking (n=30 420), and kilocalories of exercise (n=29 368).

*†*P*<0.05.

During a median follow-up of 125 months (interquartile range, 117–130 months), there were 771 cases of AF. Compared with women who reported the least global distress, worse symptoms according to MHI-5 score were not associated with risk of AF in age-adjusted or multivariable models. Table 2 shows HRs for AF in proportional-hazards models, with and without inclusion of intercurrent cardiovascular events. In both age-stratified and multivariable analyses that also included race, body mass index, hypertension, diabetes, hypercholesterolemia, smoking, alcohol intake, kilocalories from exercise, randomized treatment assignment, and antidepressant use, there was no statistically significant association between MHI-5 category and AF risk (*P*≥0.34 for models that included intercurrent cardiovascular events; *P*≥0.15 when intercurrent events were censored). When the MHI-5 score was analyzed as a continuous variable, there remained no evidence for a linear trend (*P*=0.79 in age-stratified models) or for a nonlinear relationship in regression spline analyses (data not shown).

**Table 2.** Long-Term Relative Risks (95% CIs) of AF Among 30 746 Women Without Prior Cardiovascular Disease or AF According to MHI-5 Category

	MHI-5 Category				
	86–100 (Referent)	76–85	53–75	0–52	*P* for Trend (Continuous)
Intercurrent cardiovascular events not censored					

No. cases	359	235	129	48	

Age stratified	1.0	0.86 (0.73–1.02)	0.91 (0.74–1.11)	1.08 (0.80–1.47)	0.61

Multivariable	1.0	0.87 (0.73–1.03)	0.89 (0.72–1.09)	0.99 (0.72–1.35)	0.34

Intercurrent cardiovascular events censored					

No. cases	346	223	118	43	

Age stratified	1.0	0.85 (0.72–1.01)	0.86 (0.70–1.06)	1.01 (0.74–1.39)	0.27

Multivariable	1.0	0.86 (0.72–1.02)	0.85 (0.69–1.06)	0.92 (0.66–1.29)	0.15

Multivariable models included age, race, body mass index, hypertension, diabetes, hypercholesterolemia, smoking, alcohol intake, kilocalories from exercise, randomized treatment assignment, and antidepressant use. Because of missing data, the total number of observations was lower for multivariable models (n=29 983).

These results did not materially change when we combined the data from the MHI-5 score with information on antidepressant use by creating a proxy variable for depression, consisting of MHI-5 score <53, antidepressant use, or both. This measure was not associated with risk of AF in age-stratified (HR=1.09; 95% CI, 0.87–1.38; *P*=0.44) or multivariable (HR=0.99; 95% CI, 0.78–1.25; *P*=0.93) models that included age, race, body mass index, hypertension, diabetes, hypercholesterolemia, smoking, alcohol intake, kilocalories from exercise, and randomized treatment assignment. Models that focused on MHI-5 <53 and antidepressant use separately also did not show associations with risk of AF in age-adjusted or multivariable analyses. Censoring intercurrent cardiovascular events did not materially affect our HR estimates for depression. In addition, we did not detect statistically significant relationships between MHI-5 score and AF risk in proportional-hazards models of subgroups according to race, hypertension, or diabetes.

In post hoc analyses, we analyzed the relationship between individual affective symptoms and AF risk (Table 3). In age-stratified proportional-hazards models with frequency of the symptom as an ordinal variable, none of the symptoms was linearly associated with risk of AF in age-stratified models (*P*≥0.09). When we analyzed the frequency of individual symptoms as a categorical variable, the age-stratified HR for AF was reduced in women who reported feeling happy some/a good bit of the time (HR=0.69; 95% CI, 0.49–0.99; *P*=0.04), or most/all of the time (HR=0.68; 95% CI, 0.49–0.94; *P*=0.02). These results were not attenuated in multivariable analyses. None of the other symptoms was associated with AF risk.

**Table 3.** Crude Risk of AF in Follow-Up and Age-Stratified HRs for AF According to Specific Items on MHI-5 (N=30 746)

Item	No. Women (%)	Percentage With AF in Follow-Up	Age-Stratified HR (95% CI)
How often have you felt happy?			

None/little of the time	1197 (3.9)	3.3	Referent

Some/a good bit of the time	7148 (23.3)	2.2	0.69 (0.49–0.99)

Most/all of the time	22 401 (72.9)	2.6	0.68 (0.49–0.94)

How often have you felt downhearted and blue?			

None/little of the time	25 456 (82.8)	2.6	Referent

Some/a good bit of the time	4893 (15.9)	2.2	1.01 (0.82–1.24)

Most/all of the time	397 (1.3)	1.8	0.76 (0.36–1.60)

How often have you been a very nervous person?			

None/little of the time	25 029 (81.4)	2.6	Referent

Some/a good bit of the time	5242 (17.1)	2.0	0.90 (0.73–1.11)

Most/all of the time	475 (1.5)	1.9	0.90 (0.47–1.75)

How often have you felt so down nothing could cheer you up?			

None/little of the time	27 759 (90.3)	2.5	Referent

Some/a good bit of the time	2713 (8.8)	2.3	1.08 (0.83–1.40)

Most/all of the time	274 (0.9)	1.8	0.82 (0.34–1.99)

How often have you felt calm and peaceful?			

None/little of the time	2437 (7.9)	2.3	Referent

Some/a good bit of the time	11 832 (38.5)	2.1	0.92 (0.68–1.23)

Most/all of the time	16 477 (53.6)	2.9	1.00 (0.75–1.32)

## Discussion

In this analysis of a large cohort of women without cardiovascular disease, history of stroke, or prior AF, a composite global measure of psychological distress was not associated with AF risk. With 771 AF events, to our knowledge, ours is the largest sample of women in whom the relationship between affective symptoms and AF has been studied. Our analysis adds to the nascent evidence base on psychological symptoms and AF incidence. Lange and colleagues^8^ showed in a group of 54 patients with persistent AF that depressed mood was associated with greater risk of recurrence after DC cardioversion. In a triggering analysis that used event monitoring and electronic diaries, Lampert and colleagues^9^ observed in 75 patients with paroxysmal or persistent AF that arrhythmia episodes were more likely to be preceded by negative emotions and less likely by happiness. Our analysis indicates that psychological distress is not a long-term predictor for AF in women without known heart disease.

The lack of an association between a proxy measure for depression (consisting of MHI-5 score <53, antidepressant use, or both) and AF contrasts with previous findings of an association between this measure and risk of sudden cardiac death in women without known heart disease.^5^ One possible reason for this null result may involve differences in the effects of the autonomic nervous system on atrial versus ventricular arrhythmias. Although sympathetic activation is a well-recognized trigger for ventricular arrhythmias and sudden cardiac death,^22^ a substantial proportion of AF appears to be initiated by parasympathetic triggers, both in animal models^23^ and in clinical settings.^24^ In fact, increased heart rate variability, which reflects a predominance of parasympathetic versus sympathetic tone, has been associated with higher risk of recurrent AF after cardioversion.^25^ Depression has reproducibly been associated with reduced heart rate variability in patients with coronary artery disease,^26^ and therefore decreased cardiac parasympathetic modulation might actually reduce the risk of arrhythmia in certain individuals who are susceptible to AF. It is also possible that the null result in our study may be related to a gender difference in psychological predictors for AF. For instance, findings from the Framingham Offspring Study showed that baseline levels of tension, anger, and hostility predicted increased 10-year risk of AF in men but not in women.^27,28^

When we examined individual items from the MHI-5 that assessed different types of affect, none of the items that measured negative affect was associated with AF risk. Nevertheless, in a hypothesis-generating finding, reported happiness some or more of the time was associated with reduced AF risk. A recent meta-analysis of samples of healthy patients estimated a reduced risk of all-cause and cardiac death associated with positive affect, independent of negative affect.^29^ Our finding of reduced AF risk in association with greater reported positive affect is intriguing but requires confirmation in prospective studies.

Several limitations to this study should be mentioned. We relied on a 5-item self-report instrument to measure psychological distress and did not have clinical interview data on psychiatric diagnoses, such that misclassification of depressive symptoms may have biased our findings toward the null result. However, the MHI-5 has been shown to have high accuracy at identifying individuals with mood disorders and anxiety disorders^14–16^ and has previously been correlated with cardiac^5^ and other medical^20,30^ conditions in large female cohorts. In addition, we did not have repeated assessments of psychological distress, so we cannot estimate how risk of AF relates to changes in distress over time. Our assessment of AF events depended on participant self-report, with subsequent verification by medical records review. If participants with greater psychological distress were less willing to report their AF episodes, our estimates of the relationship might be biased toward a null result. In addition, screening electrocardiograms were not performed in this cohort; therefore, it is possible that asymptomatic cases of AF may have gone undetected. Nevertheless, in this cohort of health professionals, significant underdetection is less likely; a prior analysis in the Women's Health Study cohort indicated that 10% of the confirmed AF events were asymptomatic.^21^ Also, our sample was from a group of women of mostly European ethnic origin, and we cannot extrapolate these findings to men or to other ethnic groups.

In summary, in this large prospective study of women without known cardiovascular disease, a global measure of psychological distress was unrelated to AF risk. Future studies should include standardized and more detailed instruments of depression and anxiety to test for their association to AF incidence.
